# CT texture analysis of vertebrobasilar artery calcification to identify culprit plaques

**DOI:** 10.3389/fneur.2024.1381370

**Published:** 2024-05-13

**Authors:** Bo Liu, Chen Xue, Haoyu Lu, Cuiyan Wang, Shaofeng Duan, Huan Yang

**Affiliations:** ^1^Qilu Hospital, Shandong University, Jinan, Shandong, China; ^2^School of Medical Imaging, Binzhou Medical University, Binzhou, Shandong, China; ^3^Shandong Cancer Hospital and Institute, Shandong First Medical University, Tai’an, Shandong, China; ^4^Shandong Provincial Hospital Affiliated to Shandong First Medical University, Jinan, China; ^5^GE Healthcare, Shanghai, China

**Keywords:** calcification, texture analysis, radiomics, computed tomography, plaque

## Abstract

**Objectives:**

The aim of this study was to extract radiomic features from vertebrobasilar artery calcification (VBAC) on head computed tomography (CT) images and investigate its diagnostic performance to identify culprit lesions responsible for acute cerebral infarctions.

**Methods:**

Patients with intracranial atherosclerotic disease who underwent vessel wall MRI (VW-MRI) and head CT examinations from a single center were retrospectively assessed for VBAC visual and textural analyses. Each calcified plaque was classified by the likelihood of having caused an acute cerebral infarction identified on VW-MRI as culprit or non-culprit. A predefined set of texture features extracted from VBAC segmentation was assessed using the minimum redundancy and maximum relevance method. Five key features were selected to integrate as a radiomic model using logistic regression by the Aikaike Information Criteria. The diagnostic value of the radiomic model was calculated for discriminating culprit lesions over VBAC visual assessments.

**Results:**

A total of 1,218 radiomic features were extracted from 39 culprit and 50 non-culprit plaques, respectively. In the VBAC visual assessment, culprit plaques demonstrated more observed presence of multiple calcifications, spotty calcification, and intimal predominant calcification than non-culprit lesions (all *p* < 0.05). In the VBAC texture analysis, 55 (4.5%) of all extracted features were significantly different between culprit and non-culprit plaques (all *p* < 0.05). The radiomic model incorporating 5 selected features outperformed multiple calcifications [AUC = 0.81 with 95% confidence interval (CI) of 0.72, 0.90 vs. AUC = 0.61 with 95% CI of 0.49, 0.73; *p* = 0.001], intimal predominant calcification (AUC = 0.67 with 95% CI of 0.58, 0.76; *p* = 0.04) and spotty calcification (AUC = 0.62 with 95% CI of 0.52, 0.72; *p* = 0.005) in the identification of culprit lesions.

**Conclusion:**

Culprit plaques in the vertebrobasilar artery exhibit distinct calcification radiomic features compared to non-culprit plaques. CT texture analysis of VBAC has potential value in identifying lesions responsible for acute cerebral infarctions, which may be helpful for stroke risk stratification in clinical practice.

## Introduction

The prevalence of vertebrobasilar artery (VBA) calcification is high in the intracranial artery, with rates of approximately 20% in the elderly and up to 50% in stroke patients (1). The presence of intracranial artery calcification is suggestive of coexisting atherosclerosis and associated with an elevated risk of stroke (2). Increasing studies have suggested location-dependent differences in the development of atherosclerosis in the posterior circulation plaques compared to the anterior circulation plaques (3). An understanding of the characteristics of VBA calcification may aid in improving stroke risk stratification in the posterior circulation.

Calcification, as a component of advanced atherosclerotic plaque, has been suggested to be associated with ischemic vascular events in various arterial beds. Scattered and multiple calcifications were proposed to be correlated with a higher wall shear stress, thus potentially triggering embolic stroke and acute cardiovascular events (4). Intimal-predominant calcification was associated with an increased risk of stroke and plaque instability in intracranial arteries (5, 6). Nevertheless, the interpretation and analysis of calcification morphology remain restricted to the expertise of radiologists. It is essential to use advanced statistical descriptors to facilitate the non-invasive quantification of radiologic data beyond human-eye capabilities.

Radiomics involves texture analysis by extracting thousands of features to model the spatial distribution of voxel gray-level intensity (7). By applying high-order statistics to quantify image heterogeneity, texture analysis has been greatly developed in oncology (8). Recently, radiomics of coronary artery calcium outperformed the conventional Agatston score in predicting major adverse cardiovascular events on cardiac computed tomography (CT) (9). Therefore, we hypothesized that radiomics of intracranial artery calcification contain more information beyond what can be visually observed or manually quantified.

High-resolution vessel wall magnetic resonance imaging (VW-MRI) allows for the identification of intracranial atherosclerotic plaque that is responsible for recent ischemic events (10). Interpreting calcification can be challenging on routine MRI due to insufficient resolution to detect hypointensity, which is usually surrounded by soft plaque components with complex signal characteristics. CT remains the preferred modality for better visualization and characterization of calcifications. In acute stroke patients, CT is commonly used as an initial diagnostic tool for a shorter acquisition time and greater availability. Combining CT with VW-MRI images enables a comprehensive assessment of plaque calcification, which is easy to implement in the vertebrobasilar artery due to its linear trajectory above the foramen magnum. The purpose of this study was to assess the performance of CT-based texture analysis, as compared to conventional visual assessments, in identifying culprit VBA plaques using VW-MRI as a reference standard.

## Materials and methods

### Study population

The cohort for this study was drawn from patients who underwent vessel wall MRI (VW-MRI) and head computed tomography (CT) scans within a two-week period at our institution from January 2020 to October 2023. Inclusion criteria were (i) a moderate-to-severe degree of luminal stenosis (>50%) (11) in the vertebrobasilar artery (VBA) on the VW-MRI and (ii) the concurrent presence of calcification on the CT images. Exclusion criteria were (i) evidence of non-atherosclerotic intracranial vascular pathology (e.g., cardiac embolism, dissection, vasculitis, aneurysm, Moya-Moya disease), (ii) patients with a history of stent or treatment of the target vessel, (iii) inadequate image quality or insufficient imaging coverage. The requirement for informed consent was waived in this Institutional Review Board-approved study.

### MRI examination

MRI examinations were performed on a 3 T MR imaging scanner (Achieva; Philips Healthcare, Best, the Netherlands). A standardized imaging protocol included diffusion-weighted imaging (DWI), and pre-and post-contrast VW-MRI. VW-MRI was acquired by using a T1-weighted volumetric isotropic turbo spin-echo acquisition (T1-VISTA) (TR/TE, 425 ms/19 ms; acquired resolution, 0.7 × 0.7 × 1.1 mm^3^; scan time, 6.1 min). The T1-VISTA images were repeated 5 min after contrast administration of 0.1 mmol/kg contrast agent (dimeglumine gadopentetate). Luminal stenosis was measured on T1-VISTA images using criteria established in the Warfarin-Aspirin Symptomatic Intracranial Disease trial (12). A culprit plaque was identified as the only or most stenotic lesion arising in the vertebrobasilar artery territory with the corresponding presence of acute cerebral infarction (hyperintensity on the DWI images and hypointensity on the ADC images) (13), and a non-culprit plaque was deemed if it occurred in patients without acute posterior circulation cerebral infarction.

### CT imaging and analysis

The CT was conducted on a 128-slice dual-source CT scanner (SOMATOM Definition Flash, Siemens Healthcare, Forchheim, Germany). The CT scan was acquired from the foramen magnum to the top of the skull, using a tube voltage of 120 kV, a tube current of 188 mA, a section thickness of 1 mm, and a slice acquisition interval of 1 mm. Image quality was graded based on the presence and severity of artifacts (beam hardening, photon starvation and noise) using a three-point scale (poor, adequate or excellent). All the images were determined to be of adequate or excellent quality for analysis. Two radiologists independently interpreted all CT and VW-MRI images using specific anatomical landmarks (e.g., vertebrobasilar junction). The disagreement during data annotation was resolved by consulting with a senior radiologist, who had more than 10 years of experience in plaque imaging. Calcification refers to areas of hyperdensity with a CT attenuation value of 130 Housfield units (HU) or higher. Calcification visual assessments included: (1) calcification number, categorized as either single or multiple; (2) spotty calcification, defined as calcium deposits less than 3 mm in length within an arc of less than 90 degrees (14); (3) calcification morphology, classified as either intimal-predominant or non-intimal predominant based on a validated grading scale (15).

### CT texture analysis

**Figure 1:**
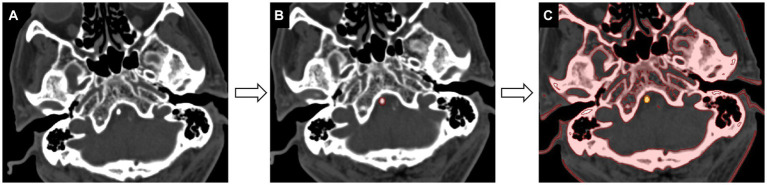
Workflow of calcification segmentation. **(A)** The acquired image is viewed at the bone window setting [window level of 300 HU (Housfield units) and window width of 1,500 HU]. **(B)** The calcification region of interest is delineated slice by slice in the axial-view images (red). **(C)** The segmentation of calcification is achieved by selecting pixels with attenuation values above 130 HU (yellow).

The processes of texture analysis were as follows:

1. Data loading: All calcification voxels accompanied by a plaque in the original CT images were converted into the Neuroimaging Informatics Technology Initiative (NIfTI) format and loaded into Medical Imaging Interaction Toolkit (MITK, open-source software, https://www.mitk.org).
2. Segmentation: The region of interest (ROI) was manually delineated along the margins of calcification slice by slice in the axial plane. The adjacent cervical vertebra was carefully excluded from the ROI. A volume of interest for each VBA calcification segmentation was created semi-automatically by selecting pixels with attenuation above 130 HU (Figure 1).
3. Feature extraction: Radiomic feature extraction was performed using an open-source Python-based tool-Pyradiomcis package (Python 3.7.9, Pyradiomics 3.0.1). The calculated radiomic features are shown in Supplementary Table S1. To obtain high-order textural features, two image pre-processing methods were employed for creating transformed-based datasets. For the Laplacian of Gaussian (LoG) filter, sigma values were set as 1, 2, 3, 4, and 5 mm to extract fine, medium, and coarse features. Wavelet filtering yields 8 decompositions including LLH, LHL, LHH, LLL, HLL, HLH, HHL, and HHH, of which H stands for high-frequency and L stands for low-frequency.
4. Feature selection: All the extracted radiomics features with statistically significant differences were selected via the minimum redundancy maximum relevance (mRMR) scheme, which allows for a joint effect of minimizing the redundancy and maximizing the relevance using fewer features. The number of features selected by mRMR algorithm was set to 5.
5. Prediction classifier: A linear combination of 5 feature variables selected by the mRMR algorithm was constructed using multivariate logistic regression method according to the Akaike Information Criteria. The predictor value in the logistic regression model was transformed into a probability for two-class prediction.

### Statistical analysis

Categorical data were presented as frequencies. For continuous variables, mean ± standard deviations were used for normal distribution, while median and interquartile range (IQR) were used for skewed distribution. The characteristics between different groups were tested using student’s t-test or Mann–Whitney U test for continuous variables, and χ^2^ test for categorical variables. The diagnostic performance of all models was evaluated using receiver operating characteristic (ROC) analysis and the DeLong test. The area under the curve (AUC), accuracy, sensitivity, specificity, positive predictive value and negative predictive value were calculated for identifying culprit plaques. Ten-fold cross-validation was used to evaluate the robustness of the model (16). A two-tailed *p* value < 0.05 was statistically significant. All statistical analyses were performed with R software (version 4.0.2).

## Results

### Patient and lesion characteristics

**Figure 2:**
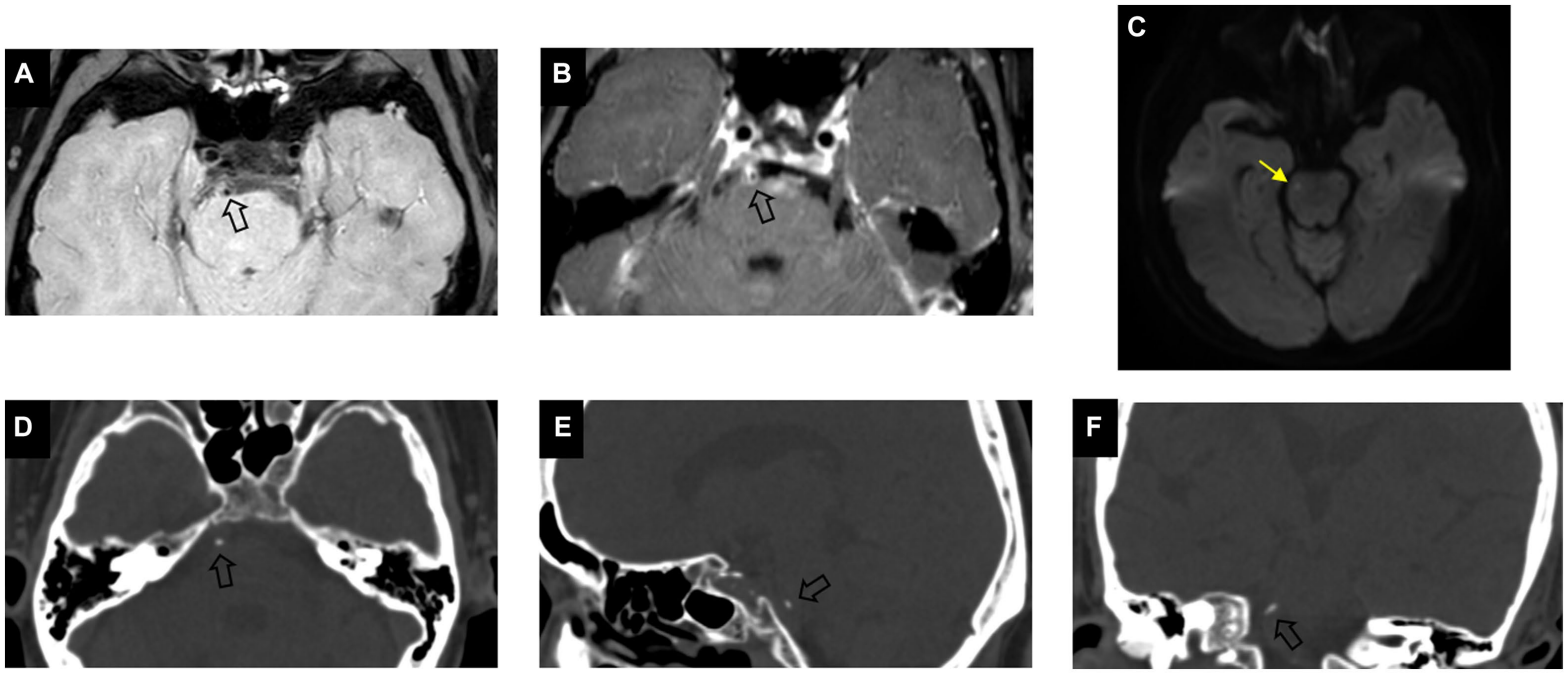
Example of calcification presence in a culprit plaque of a 64-year-old male symptomatic patient. Pre-contrast **(A)** and post-contrast **(B)** vessel wall MRI show an eccentric plaque (arrow) in the basilar artery. DWI image **(C)** demonstrates the presence of hyperintensity (yellow arrow) in the brainstem. Axial **(D)**, sagittal **(E)**, and coronal **(F)** CT images demonstrate the corresponding presence of calcification (arrow) which is classified as single, spotty, and intimal predominant calcification on visual assessment.

**Figure 3:**
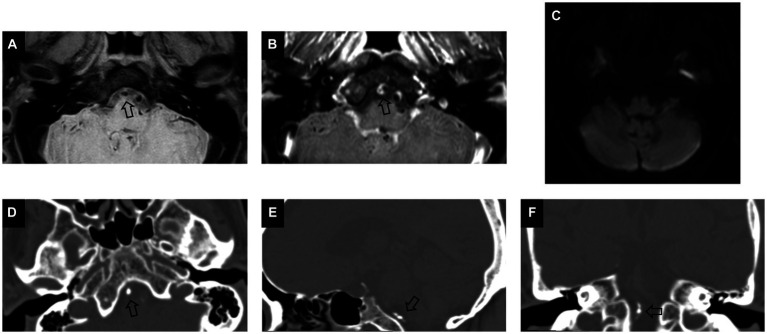
Example of calcification presence in a non-culprit plaque of a 66-year-old female asymptomatic patient. Pre-contrast **(A)** and post-contrast **(B)** vessel wall MRI images show an eccentric plaque (arrow) in V4 segment of the vertebral artery. DWI image **(C)** demonstrates no presence of hyperintensity in the posterior circulation. Axial **(D)**, sagittal **(E)**, and coronal **(F)** CT images demonstrate the corresponding presence of calcification (arrow), which is classified as multiple, non-spotty, and non-intimal predominant calcification on visual assessment.

A total of 102 patients were eligible for the study based on the inclusion and exclusion criteria. Eight patients with acute cerebral infarction and five without acute cerebral infarction were excluded due to incomplete high-order information from ROI segmentation. Finally, a total of 89 patients (59 male; mean age, 62.7 ± 8.4 years) were included in this study, of which 39 had acute cerebral infarction. As shown in Table 1, there were no significant differences in clinical characteristics between the two groups. Compared to non-culprit plaques, culprit plaques had a higher incidence of basilar artery calcification (*p* = 0.004) and a greater degree of stenosis (*p* = 0.032). In the visual assessment of calcification characteristics, culprit plaques showed a higher prevalence of multiple calcifications (28% vs. 6%, *p* = 0.007), intimal predominant calcification (46% vs. 12%, *p* < 0.001), and spotty calcification (54% vs. 30%, p = 0.03) compared to non-culprit plaques. Figures 2, 3 present the representative CT and VW-MRI images for the culprit and non-culprit lesions, respectively.

**Table 1:** Patients and lesion characteristics.

	ACI	Non-ACI	*P*-value
	(*N* = 39)	(*N* = 50)	
Age, year	62.77 ± 9.36	62.68 ± 7.70	0.961
Male	26 (67%)	33 (66%)	1.000
BMI, kg/m^2^	27.75 ± 2.98	26.97 ± 3.65	0.401
**Risk factors**			
Diabetes mellitus	27 (69%)	30 (60%)	0.385
Smoking	13 (33%)	10 (20%)	0.222
Hypertension	36 (92%)	47 (94%)	1.000
Hyperlipidemia	18 (46%)	21 (42%)	0.830
Coronary heart disease	18 (46%)	17 (34%)	0.279
**Drug use**			
Cholesterol-lowering	5 (13%)	13 (25%)	0.184
Antiplatelet	8 (21%)	15 (30%)	0.340
Time interval between vw-MRI and CT	2 (1, 4)	4 (2, 7)	0.173
Infarction locations	64		
Medulla	5 (8%)		
Pons	15 (23%)		
Midbrain	1 (2%)		
Cerebellum	24 (37%)		
Occipital lobe	12 (19%)		
Temporal lobe	3 (5%)		
Thalamus	2 (3%)		
Callosum	2 (3%)		
Plaque location			0.004^*^
Basilar artery	10 (26%)	2 (4%)	
Vertebral artery	29 (74%)	48 (96%)	
Stenosis degree (%)	68.29 (9.20)	61.94 (12.32)	0.032^*^
Calcification characteristics			
Number			0.007^*^
Single	28 (72%)	47 (94%)	
Multiple	11 (28%)	3 (6%)	
Spotty calcification			0.030^*^
Negative	18 (46%)	35 (70%)	
Positive	21 (54%)	15 (30%)	
Intimal predominant calcification			<0.001^*^
Negative	21 (54%)	44 (88%)	
Positive	18 (46%)	6 (12%)	

Values are mean ± SD or number (%). ^*^p < 0.05. ACI, acute cerebral infarction; BMI, body mass index; CT, computed tomography; vw-MRI, vessel wall magnetic resonance imaging.

### Radiomic analysis of calcification features for identifying culprit plaques

**Figure 4:**
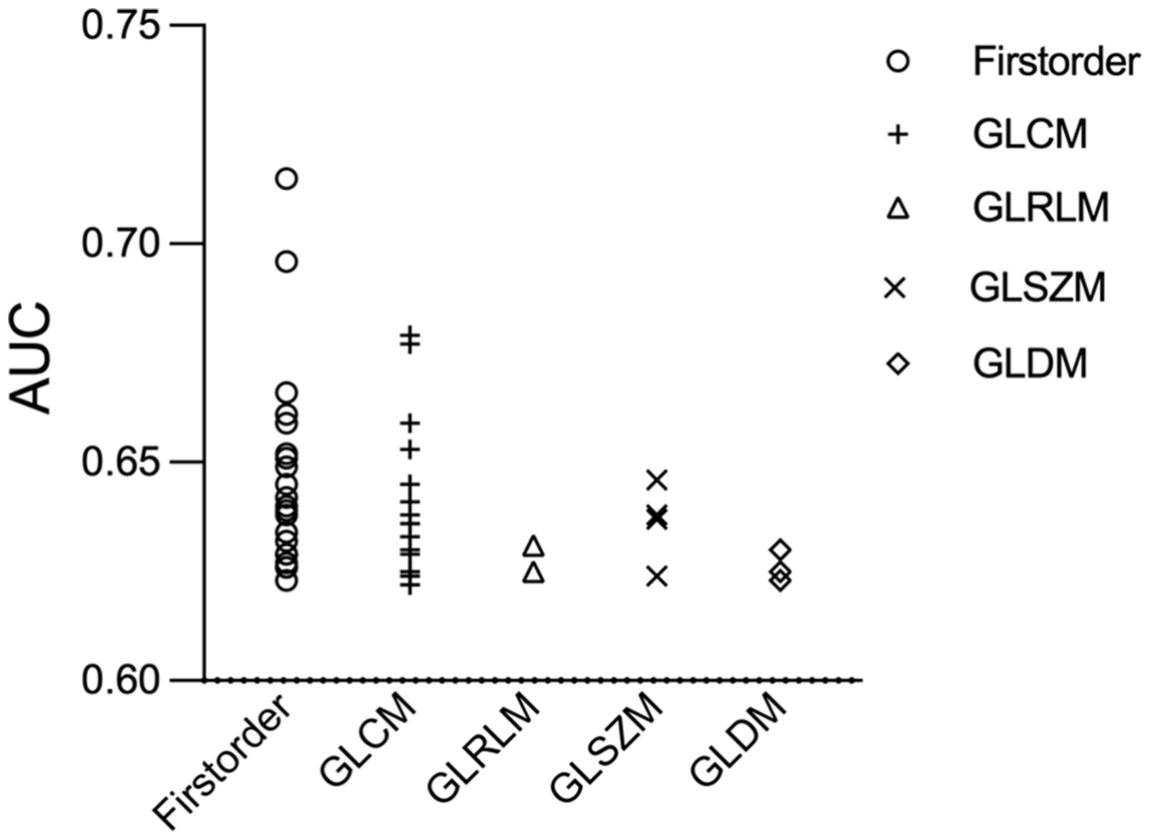
Receiver operating characteristic scatter plot of 55 radiomics features. Radiomic parameters are situated on the x-axis, while their corresponding AUC values to identify culprit lesions are shown on the y-axis. AUC, the area under the curve; GLCM, gray level co-occurrence matrix; GLDM, gray-level dependence matrix; GLRLM, gray level run length matrix; GLSZM, gray-level size zone matrix.

Out of the 1,218 extracted features, 55 transform-based features showed a significant difference between culprit and non-culprit lesions (Supplementary Table S2). Among them, 22 (40.0%) were first order, 24 (43.6%) were gray level co-occurrence matrix (GLCM), 3 (5.5%) were gray-level dependence matrix (GLDM), 2 (3.6%) were gray level run length matrix (GLRLM) and 4 (7.3%) were gray-level size zone matrix (GLSZM) parameters (Figure 4). All the 55 calculated statistics yielded an AUC higher than 0.60.

**Figure 5:**
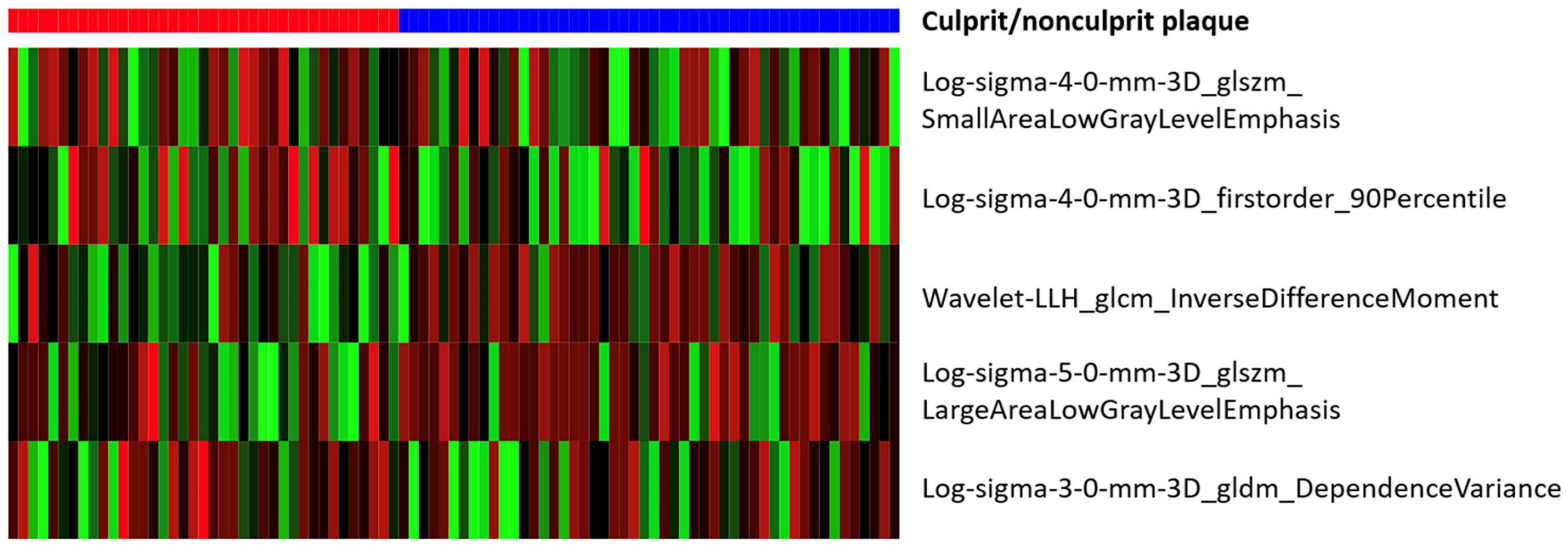
Heat map of associations between the 5 radiomics features and culprit lesions.

After mRMR selection, 5 features (Table 2) were retained to construct the optimal radiomic signature, which encompassed 4 features in the LoG-filtered image and 1 feature in the wavelet-filtered image. The association between the 5 radiomics features and culprit lesions is shown in Figure 5. A logistic regression model was built with the 5 radiomics features and defined the importance levels.

**Table 2:** Multivariate logistic regression analyses of the radiomics features for identifying culprit plaques.

Variables	OR	95%CI	*P*-value
log-sigma-4-0-mm-3D_glszm_SmallAreaLowGrayLevelEmphasis	0.73	0.40, 1.32	0.295
log-sigma-5-0-mm-3D_glszm_LargeAreaLowGrayLevelEmphasis	1.49	0.73, 3.04	0.278
wavelet-LLH_glcm_InverseDifferenceMoment	3.34	1.27, 8.81	0.015
log-sigma-3-0-mm-3D_gldm_DependenceVariance	0.47	0.23, 0.95	0.036
log-sigma-4-0-mm-3D_firstorder_90Percentile	0.56	0.32, 0.99	0.045

CI, confident interval; OR, odds ratio.

**Figure 6:**
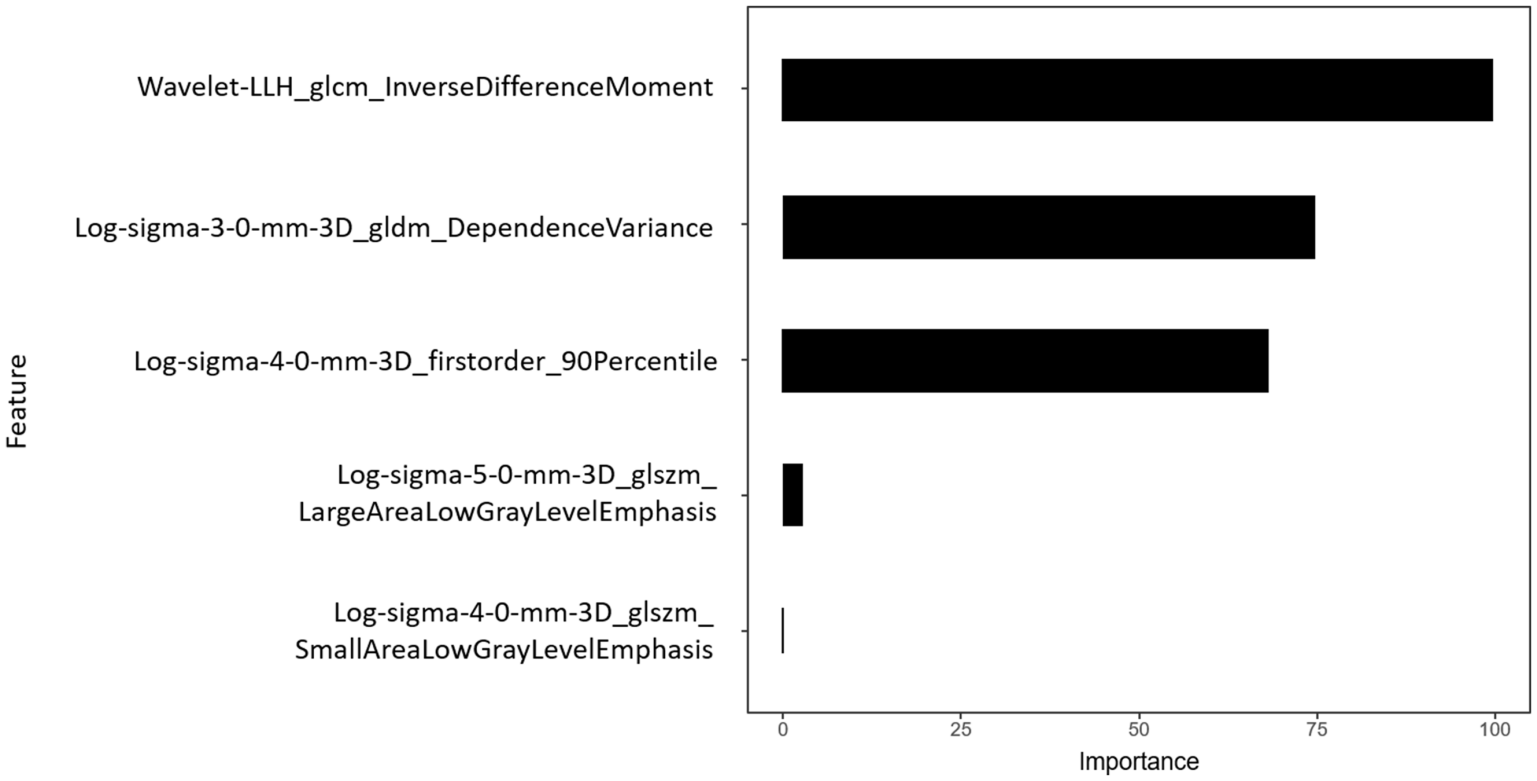
Importance of radiomics features. Histogram showed the role of the final five selected features that contribute to the radiomics signature.

**Figure 7:**
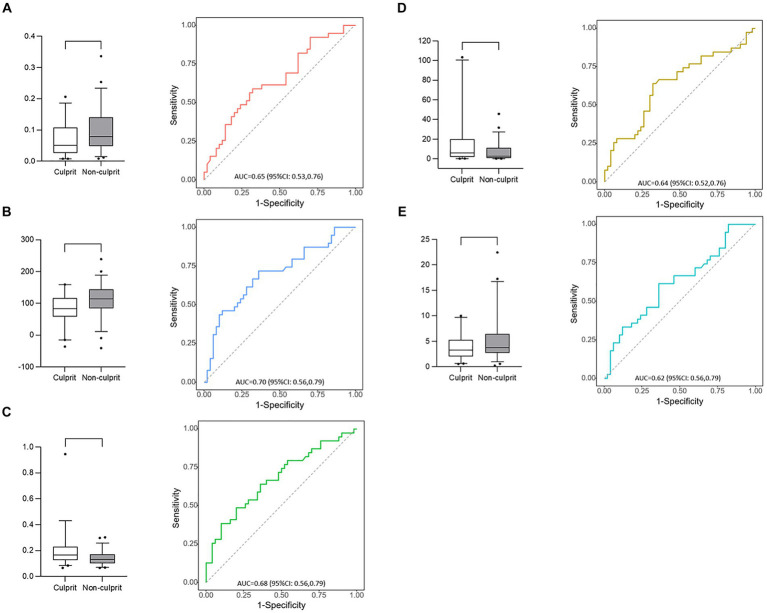
Box plots and receiver operating characteristic curves of 5 radiomic features. **(A)** Log-sigma-4-0-mm-3D_glszm_SmallAreaLowGrayLevelEmphasis; **(B)** Log-sigma-4-0-mm-3D_firstorder_90Percentile; **(C)** Wavelet-LLH_glcm_InverseDifferenceMoment; **(D)** Log-sigma-5-0-mm-3D_glszm_LargeAreaLowGrayLevelEmphasis; **(E)** Log-sigma-3-0-mm-3D_gldm_DependenceVariance.

LLH_GLCM_InverseDifferenceMoment in wavelet-filtered image was identified as the most important feature, followed by GLDM_DependenceVariance in 3 mm-sigma LoG-filtered image, firstorder_90Percentile in 4 mm-sigma LoG-filtered image, GLSZM_LargeAreaLowGrayLevelEmphasis in 5 mm-sigma LoG-filtered image and GLSZM_SmallAreaLowGrayLevelEmphasis in 4 mm-sigma LoG-filtered image (Figure 6). Among the 5 features, Log-sigma-4-0-mm-3D_firstorder_90Percentile showed the highest AUC value [0.70; 95% confidence interval (CI): 0.58–0.81] (Figure 7).

### Comparison of diagnostic performance of conventional and radiomics models

**Figure 8:**
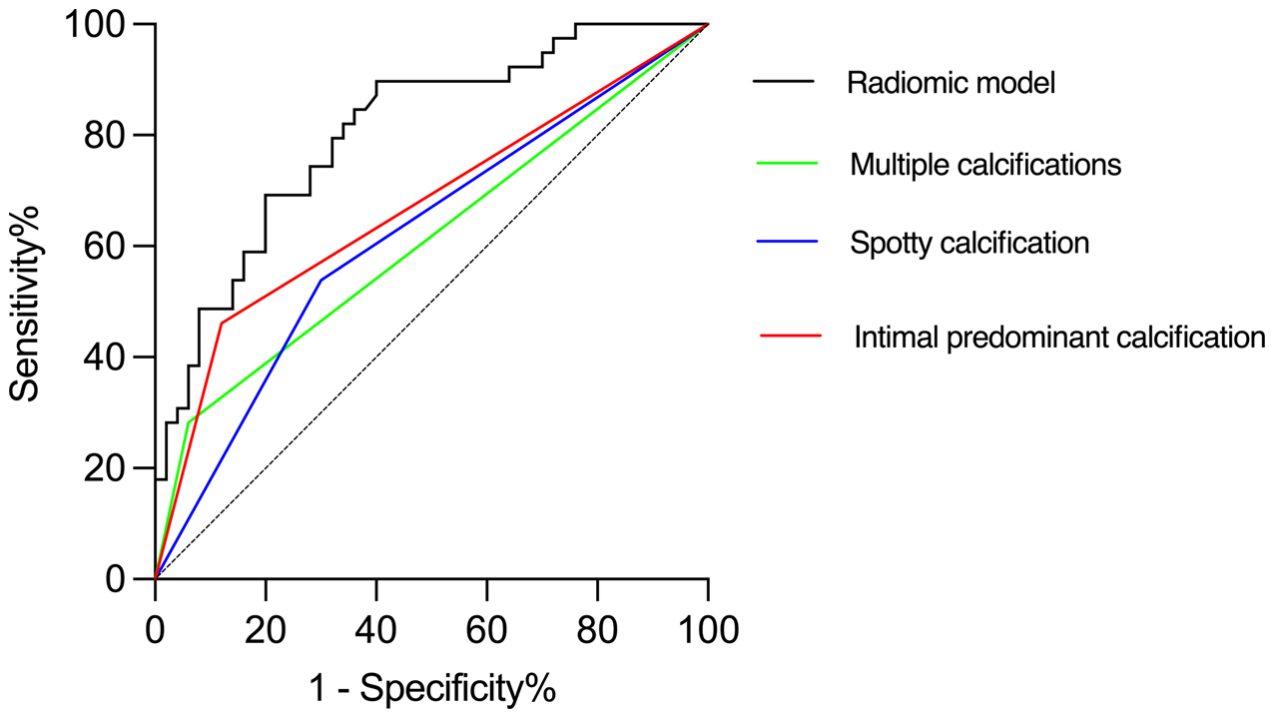
Receiver operating characteristic curves of radiomics model and visual assessments (multiple calcifications, spotty calcification and intimal predominant calcification) in the identification of culprit lesions. Radiomics model showed the best discriminatory power [the area under the curve (AUC) = 0.81, 95% confidence interval (CI): 0.72–0.90]. The discriminatory power of visual assessment with use of multiple calcifications (AUC = 0.61, 95% CI: 0.49–0.73; *P* = 0.001), spotty calcification (AUC = 0.62, 95% CI: 0.52–0.72; *P* = 0.005) and intimal predominant calcification (AUC = 0.67, 95% CI: 0.58–0.76; *P* = 0.04) showed poor diagnostic accuracy compared with the radiomics model.

The diagnostic performances of the conventional and radiomics models are shown in Table 3. As shown in Figure 8, the radiomics model demonstrated significantly higher AUC for the detection of culprit plaques (0.81; 95% CI: 0.72–0.90) compared with the presence of multiple calcifications (0.61; 95% CI: 0.49–0.73, *p* = 0.001), spotty calcification (0.62; 95% CI: 0.52–0.72, *p* = 0.005), and intimal predominant calcification (0.67; 95% CI: 0.58–0.76, *p* = 0.04). Ten-fold cross-validation was conducted to demonstrate the model’s robustness, yielding a mean AUC of 0.76.

**Table 3:** Diagnostic performance of conventional models and radiomics models to identify culprit plaques.

Models	AUC (95%CI)	Accuracy	Sensitivity	Specificity	PPV	NPV
Multiple calcifications	0.61 (0.49–0.73)	0.65	0.28	0.94	0.78	0.63
Spotty calcification	0.62 (0.52–0.72)	0.63	0.70	0.54	0.66	0.58
Intimal calcification	0.67 (0.58–0.76)	0.70	0.88	0.46	0.68	0.75
Radiomic model	0.81 (0.72–0.90)	0.73	0.60	0.90	0.88	0.64

AUC, area under the curve; CI, confidence interval; NPV, negative predict value; PPV, positive predict value.

## Discussion

In this study, we performed a three-dimensional texture analysis to determine whether CT-based radiomic features could be used to discriminate culprit lesions. We also intended to evaluate the diagnostic performance of texture analysis from VBAC compared with conventional methods. Our study found multiple calcification texture parameters differed significantly between culprit and non-culprit lesions. A model with a subset of 5 features according to the minimal-redundancy-maximal-relevance criterion achieved an AUC of 0.81 and an accuracy of 0.73, which significantly outperformed VBAC visual assessments.

To our knowledge, this was the first study to assess the association between radiomic features of intracranial artery calcification and plaque instability using multi-modality imaging methods. Several MRI-defined vulnerable plaque characteristics, such as intraplaque hemorrhage (17) and lipid-rich necrotic core (18), have been validated by specimens from carotid endarterectomy. Nevertheless, current challenges in MR imaging intensity features of intracranial plaque components included spatial resolution and histopathologic validation (19). Vessel wall calcification can be objectively detected on CT images owing to its high attenuation (20). CT texture analysis of vertebrobasilar artery calcification may serve as a promising imaging marker of plaque instability, thereby assisting in clinical decision-making and improving patient outcomes in the posterior circulation stroke.

Several studies have investigated the visual assessment of plaque calcification in relation to stroke risk. Multiple calcifications were associated with the presence of carotid intraplaque hemorrhage, therefore implicating an increased plaque vulnerability (21, 22). A higher prevalence of spotty calcification in the cervicocerebral artery was reported in stroke patients compared with controls (14). A study has found that spotty calcification in nonstenotic carotid atherosclerosis is associated with ischemic stroke on the same side (4). Other studies have demonstrated the pattern of calcification within the intimal layer was associated with unstable plaque phenotype (6) and increased stroke risk (5). In the current study, multiple calcifications had a low sensitivity of 28%, and spotty calcification and intimal predominant calcification have relatively low specificity (54 and 46%) for the presence of culprit lesions. These performances may be explained by the simultaneous development of atherosclerotic calcification in the intimal layer and non-atherosclerotic calcification in the internal elastic lamina and medial layers. Hence, visual assessments of calcification based on its morphology may not provide sufficient data to determine plaque instability.

Radiomics uses computational methods to extract thousands of quantitative features for comprehensive calcification description, mostly imperceptible to the human eye (23). In the animal model of hyperlipidemic mice, several radiomic features of vascular calcium were associated with aging and Western diet, which cannot be determined from calcium scores from conventional CT or total calcium content (24). In the clinical setting, a radiomic-based model was developed from coronary artery calcium radiomic features, resulting in significant improvement in predicting individuals at risk for clinical events (9). In line with previous research, our study demonstrates the significance of calcification radiomics features that extend beyond conventional visual evaluations, achieving the highest AUC values. The diagnostic performance of texture analysis in identifying culprit lesions exhibited a specificity of 90%. This high level of true negative rate suggests that the radiomic model may be better at identifying non-culprit lesions than culprit ones.

Of the 5 selected features, LLH_GLCM_InverseDifferenceMoment in wavelet-filtered image, GLDM_DependenceVariance in 3 mm-sigma LoG-filtered image, and firstorder_ 90Percentile in 4 mm-sigma LoG-filtered image have the significant effect on the radiomic model. The lower value of firstorder_90Percentile in the culprit lesion implies a possible association between low calcification density and plaque instability. GLCM_InverseDifferenceMoment is a measure used to evaluate the homogeneity of an image. GLDM_DependenceVariance is a metric that measures the degree of asymmetry in the distribution of values around the mean value. The above two features suggest that calcification within culprit plaques is more uniform and homogeneous. These variations in texture analysis may offer insights into the underlying pathophysiology of calcium deposits. The lesion, consisting primarily of a specific calcium salt, may demonstrate greater homogeneity compared to the mixed type and is associated with the unstable plaque phenotype (25).

Consistent with previous studies, calcification presence in the proximal intracranial artery (e.g., basilar artery) is relatively rare compared with the distal one (e.g., vertebral artery) (26). Moreover, a higher portion of basilar artery calcification was present in the culprit lesions than in the non-culprit ones. A significant discrepancy was observed in the pattern of calcification within the intracranial arteries (27). A previous study found that calcification in the middle cerebral artery is less pronounced on the symptomatic side compared to the asymptomatic side on CT images (26). In the intracranial internal carotid artery, no difference was detected in the presence of calcification between the symptomatic and asymptomatic sides (28). In a study involving seven intracranial arteries, the presence of calcification is associated with mortality and vascular events in ischemic stroke patients (29). A larger sample size would be necessary to further investigate the relationship between basilar artery calcification and plaque instability.

This study has several limitations. First, the study is retrospective, and the possibility of selection bias cannot be avoided. Second, the sample size of enrolled patients was limited. Texture analysis exhibits significant sensitivity to technical factors, including CT acquisition parameters (30). Therefore, all CT scans in our study were conducted using the same scanning to prevent bias in the extraction of radiomics features. Third, the ROI of calcification is small. We utilized a three-dimensional texture analysis to assess calcification texture, which provided superior performance compared to a two-dimensional approach (31). Fourth, the mRMR algorithm may be overfitted due to the absence of a distinct validation cohort. Despite this, we employed a tenfold cross-validation technique to assess the classifier’s performance (16), improving the generalizability of the model.

## Conclusion

CT texture analysis provides an objective evaluation of calcification heterogeneity and outperforms visual assessments in identifying culprit plaques, thereby serving as a potential predictor for plaque instability in the vertebrobasilar artery.

## Data availability statement

The original contributions presented in the study are included in the article/Supplementary material, further inquiries can be directed to the corresponding author.

## Ethics statement

The studies involving humans were approved by Shandong Provincial Hospital Affiliated to Shandong First Medical University. The studies were conducted in accordance with the local legislation and institutional requirements. Written informed consent from the patients/participants or patients/participants’ legal guardian/next of kin was not required to participate in this study in accordance with the national legislation and the institutional requirements.

## Author contributions

BL: Investigation, Methodology, Writing – original draft. CX: Formal analysis, Software, Writing – original draft. HL: Resources, Visualization, Writing – original draft. CW: Data curation, Project administration, Writing – original draft, Writing – review & editing. SD: Formal analysis, Methodology, Software, Writing – original draft. HY: Conceptualization, Funding acquisition, Resources, Supervision, Writing – review & editing.
