# Supplementary material for: CT texture analysis of vertebrobasilar artery calcification to identify culprit plaques
Source: Front Neurol. 2024 May 13;15:1381370. doi: 10.3389/fneur.2024.1381370 (PMC11128659; doi:10.3389/fneur.2024.1381370)
Supplement: Supplementary file 1 [file Table_1.DOCX]

**SUPPLEMENTARY MATERIAL**

**Table S1. Overview of All Computed Radiomic Parameters on the Original Image**

| **Texture Metric** | **Texture Category** | **Meaning** | **Number** | **Texture Feature** |
| --- | --- | --- | --- | --- |
| First-order features | Histogram | Characterizing the Hounsfield unit distribution of lesions | 18 | Energy, Total Energy, Entropy, Minimum, Maximum, Mean, Median, Interquartile Range, Range, Mean Absolute Deviation, Robust Mean Absolute Deviation, Root Mean Squared, Variance, Skewness, Kurtosis, 10Percentile, 90Percentile, Uniformity |
|  |  |  |  |  |
| High-order features | Gray level co-occurrence matrix (GLCM) | Characterizing the frequency of adjacent occurrences of a particular voxel pair | 22 | Autocorrelation, Joint Average, Cluster Prominence, Cluster Shade, Cluster Tendency, Contrast, Correlation, Difference Average, Difference Entropy, Difference Variance, Joint Energy, Joint Entropy, Informational Measure of Correlation 1, Informational Measure of Correlation 2, Inverse Difference Moment, Inverse Difference Moment Normalized, Inverse Difference, Inverse Difference Normalized, Inverse Variance, Maximum Probability, Sum Entropy, Sum of Squares |
|  |  |  |  |  |
|  | Gray level run length matrix (GLRLM) | Characterizing the frequency of adjacent voxels with identical values within a specified direction | 16 | Short Run Emphasis, Long Run Emphasis, Gray Level Non-Uniformity, Gray Level Non-Uniformity Normalized, Run Length Non-Uniformity, Run Length Non-Uniformity Normalized, Run Percentage, Gray Level Variance, Run Variance, Run Entropy, Low Gray Level Run Emphasis, High Gray Level Run Emphasis, Short Run Low Gray Level Emphasis, Short Run High Gray Level Emphasis, Long Run Low Gray Level Emphasis, Long Run High Gray Level Emphasis |
|  |  |  |  |  |
|  | Gray level size zone matrix (GLSZM) | Characterizing the spatial distribution patterns of adjacent voxels | 16 | Small Area Emphasis, Large Area Emphasis, Gray Level Non-Uniformity, Gray Level Non-Uniformity Normalized, Size-Zone Non-Uniformity, Size-Zone Non-Uniformity Normalized, Zone Percentage, Gray Level Variance, Zone Variance, Zone Entropy, Low Gray Level Zone Emphasis, High Gray Level Zone Emphasis, Small Area Low Gray Level Emphasis, Small Area High Gray Level Emphasis, Large Area Low Gray Level Emphasis, Large Area High Gray Level Emphasis |
|  |  |  |  |  |
|  | Gray level dependence matrix (GLDM) | Characterizing the angular independence of features within a voxel and its neighboring voxels | 14 | Small Dependence Emphasis, Large Dependence Emphasis, Gray Level Non-Uniformity, Dependence Non-Uniformity, Dependence Non-Uniformity Normalized, Gray Level Variance, Dependence Variance, Dependence Entropy, Low Gray Level Emphasis, High Gray Level Emphasis, Small Dependence Low Gray Level Emphasis, Small Dependence High Gray Level Emphasis, Large Dependence Low Gray Level Emphasis, Large Dependence High Gray Level Emphasis |
|  |  |  |  |  |
| Shape-based features |  | Characterizing the shape and size of lesions | 14 | Mesh Volume, Voxel Volume, Surface Area, Surface Area to Volume ratio, Sphericity, Compactness 1/2, Spherical Disproportion, Maximum 3D/2D Diameter, Major/Minor Axis Length, Least Axis Length, Elongation, Flatness |

**Table S2. Texture Features with A Significant Difference between Culprit Plaques and Non-culprit Plaques**

| **Texture Features** | **Culprit Plaques （n=39）** | **Non**-**culprit Plaques （n=50）** | **P**  **Value** |
| --- | --- | --- | --- |
| log-sigma-1-0-mm-3D_  firstorder_MeanAbsoluteDeviation | 56.04  [41.89,107.17] | 82.36  [58.37,113.01] | 0.042 |
| log-sigma-1-0-mm-3D_  firstorder_Variance | 6,499.52 [2,972.50, 17,497.42] | 10,978.10 [5,149.98, 19,218.25] | 0.037 |
| log-sigma-1-0-mm-3D_  glcm_Contrast | 14.76  [8.43,41.54] | 33.67  [13.49,64.01] | 0.029 |
| log-sigma-1-0-mm-3D_  glcm_DifferenceAverage | 3.14 [2.29,5.11] | 4.51 [2.94,6.23] | 0.033 |
| log-sigma-1-0-mm-3D_  glcm_DifferenceVariance | 6.04 [2.50,13.56] | 11.78 [4.81,21.60] | 0.028 |
| log-sigma-1-0-mm-3D_  glcm_InverseDifference | 0.38 [0.27,0.43] | 0.30 [0.25,0.37] | 0.043 |
| log-sigma-1-0-mm-3D_  glcm_InverseDifferenceMoment | 0.29 [0.17,0.35] | 0.22 [0.16,0.27] | 0.046 |
| log-sigma-1-0-mm-3D_glcm_  InformationalMeasureofCorrelation2 | 0.98 [0.95,0.99] | 0.99 [0.98,1.00] | 0.026 |
| log-sigma-1-0-mm-3D_  glcm_InverseVariance | 0.29 [0.19,0.35] | 0.21 [0.16,0.29] | 0.050 |
| log-sigma-1-0-mm-3D_  glcm_SumSquares | 10.57[4.29,27.22] | 20.46 [7.86,34.90] | 0.050 |
| log-sigma-1-0-mm-3D_  gldm_GrayLevelVariance | 10.40[4.69,28.06] | 18.39 [8.33,31.12] | 0.036 |
| log-sigma-1-0-mm-3D_  glrlm_GrayLevelVariance | 10.77[4.87,28.24] | 18.73 [8.49,31.32] | 0.035 |
| log-sigma-1-0-mm-3D_  glszm_GrayLevelVariance | 12.55  [5.33,30.90] | 21.15  [11.03,35.12] | 0.026 |
| log-sigma-3-0-mm-3D_  firstorder_90Percentile | 74.37 [33.53,110.96] | 96.38 [75.67,125.91] | 0.022 |
| log-sigma-3-0-mm-3D_  firstorder_Kurtosis | 2.44 [2.08,2.93] | 2.76 [2.43,3.16] | 0.048 |
| log-sigma-3-0-mm-3D_  firstorder_Maximum | 109.40 [69.52,149.13] | 144.83 [103.28,176.15] | 0.004 |
| log-sigma-3-0-mm-3D_ firstorder_Mean | 22.50  [-31.88,59.64] | 45.28  [24.03,66.34] | 0.033 |
| log-sigma-3-0-mm-3D_ firstorder_RootMeanSquared | 1,024.34  [969.99,1,060.78] | 1,045.84 [1,026.85, 1,066.62] | 0.031 |
| log-sigma-3-0-mm-3D_ gldm_DependenceVariance | 3.25 [2.07,5.05] | 3.77 [2.78,6.22] | 0.047 |
| log-sigma-4-0-mm-3D_ firstorder_90Percentile | 83.623 [58.62,116.17] | 114.46 [85.82,142.97] | 0.002 |
| log-sigma-4-0-mm-3D_ firstorder_Maximum | 102.69 [78.02,135.77] | 138.54 [109.60,176.12] | 0.001 |
| log-sigma-4-0-mm-3D_ firstorder_Mean | 36.85  [-17.03,66.16] | 60.65  [36.69,84.36] | 0.016 |
| log-sigma-4-0-mm-3D_ firstorder_Median | 39.99  [-20.32,74.76] | 64.22  [40.49,87.46] | 0.025 |
| log-sigma-4-0-mm-3D_ firstorder_RootMeanSquared | 1,037.68  [983.77,1,066.50] | 1,060.96 [1,037.32, 1,085.57] | 0.015 |
| log-sigma-4-0-mm-3D_glszm_  SmallAreaLowGrayLevelEmphasis | 0.05 [0.03,0.10] | 0.08 [0.05,0.14] | 0.019 |
| log-sigma-5-0-mm-3D_ firstorder_90Percentile | 80.92 [41.88,109.45] | 98.91 [78.11,131.90] | 0.015 |
| log-sigma-5-0-mm-3D_ firstorder_Maximum | 98.28 [68.92,120.69] | 116.86 [100.29,151.73] | 0.007 |
| log-sigma-5-0-mm-3D_  glcm_Contrast | 1.05 [0.52,1.90] | 1.45 [1.01,2.33] | 0.044 |
| log-sigma-5-0-mm-3D_ glcm_DifferenceAverage | 0.76 [0.49,1.04] | 0.91 [0.75,1.24] | 0.036 |
| log-sigma-5-0-mm-3D_  glcm_InverseDifference | 0.67 [0.60,0.76] | 0.63 [0.53,0.68] | 0.037 |
| log-sigma-5-0-mm-3D_  glcm_InverseDifferenceMoment | 0.65 [0.57,0.76] | 0.60 [0.48,0.66] | 0.037 |
| log-sigma-5-0-mm-3D_glszm_ LargeAreaLowGrayLevelEmphasis | 5.94 [2.04,19.23] | 2.55 [1.03,9.98] | 0.028 |
| wavelet-HHL_glcm_Correlation | -0.04  [-0.16,-0.001] | -0.02  [-0.06,0.04] | 0.044 |
| Wavelet-HLL_firstorder_10Percentile | -273.00  [-386.02,-183.34] | -392.09  [-515.59,-256.32] | 0.019 |
| wavelet-HLL_  firstorder_MeanAbsoluteDeviation | 140.83 [113.37,227.84] | 202.84 [149.29,265.33] | 0.040 |
| wavelet-HLL_firstorder_Variance | 28,285.79 [17,989.40, 75,454.87] | 68,955.51 [32,932.75, 105,010.39] | 0.042 |
| wavelet-HLL_  glcm_ClusterProminence | 34,260.13 [10,234.25,  246,565.07] | 285,781.2 [31,690.23, 626,335.99] | 0.050 |
| wavelet-HLL_glcm_ClusterTendency | 102.18 [54.01,307.96] | 282.3 [109.12,454.89] | 0.036 |
| wavelet-HLL_glcm_Correlation | 0.06 [-0.09,0.13] | 0.12 [0.01,0.24] | 0.014 |
| wavelet-HLL_glcm_SumSquares | 49.07 [28.64,142.57] | 124.07 [54.03,178.60] | 0.045 |
| wavelet-HLL_  gldm_GrayLevelVariance | 46.69 [28.76,121.42] | 110.04 [52.82,168.05] | 0.044 |
| wavelet-HLL_  glrlm_GrayLevelVariance | 46.97 [28.88,122.23] | 110.48 [52.84,168.93] | 0.043 |
| wavelet-HLL_ glszm_GrayLevelVariance | 47.53 [29.89,132.14] | 114.77 [53.35,172.23] | 0.045 |
| wavelet-LHL_glcm_Correlation | 0.09 [-0.07,0.21] | 0.18 [0.04,0.29] | 0.019 |
| wavelet-LLH_firstorder_10Percentile | -226.87  [-282.45,-152.17] | -255.16  [-368.22,-207.07] | 0.024 |
| wavelet-LLH_ firstorder_90Percentile | 126.54  [85.43,167.74] | 154.85  [113.55,234.09] | 0.026 |
| wavelet-LLH_ firstorder_InterquartileRange | 183.25  [121.00,235.07] | 227.32  [172.93,319.33] | 0.009 |
| wavelet-LLH_  firstorder_MeanAbsoluteDeviation | 115.70  [81.01,144.38] | 134.99  [106.77,189.30] | 0.038 |
| wavelet-LLH_firstorder_  RobustMeanAbsoluteDeviation | 76.19  [52.54,105.02] | 93.66  [73.13,129.38] | 0.010 |
| wavelet-LLH_glcm_ClusterTendency | 70.14  [32.43,142.15] | 95.41  [55.48, 235.14] | 0.037 |
| wavelet-LLH_  glcm_InverseDifference | 0.25 [0.22,0.31] | 0.22 [0.18,0.26] | 0.010 |
| wavelet-LLH_ glcm_InverseDifferenceMoment | 0.17 [0.13,0.23] | 0.13 [0.10,0.17] | 0.004 |
| wavelet-LLH_glcm_  InformationalMeasureofCorrelation2 | 0.99 [0.98,1.00] | 1.00 [0.99,1.00] | 0.004 |
| wavelet-LLH_glcm_InverseVariance | 0.16 [0.12,0.20] | 0.13 [0.11,0.17] | 0.023 |
| wavelet-LLL_ glcm_DifferenceVariance | 54.72  [21.97,141.53] | 124.53  [41.56,231.82] | 0.045 |
